# Deaf older adults’ experiences of receiving old age care support

**DOI:** 10.1093/jdsade/enag019

**Published:** 2026-05-11

**Authors:** Elin Karlsson, Yashar Mahmud, Susanne Andersson, Linda Jonsson, Åsa Gustavsson, Sofia Kjellström Professor, Sofi Fristedt

**Affiliations:** Audiological Research Centre, Faculty of Medicine and Health, Örebro University, Örebro, Sweden; School of Health Sciences, Faculty of Medicine and Health, Örebro University, Örebro, Sweden; School of Communication Sciences and Disorders, Western University, London, ON, Canada; School of Health and Welfare, Jönköping University, Jönköping, Sweden; Department of Leadership and Command & Control, Swedish Defence University, Stockholm, Sweden; School of Health and Welfare, Jönköping University, Jönköping, Sweden; School of Health and Welfare, Jönköping University, Jönköping, Sweden; The Association of the Deaf and Sign Language Users in Jönköping County, Jönköping, Sweden; School of Health and Welfare, Jönköping University, Jönköping, Sweden; School of Health and Welfare, Jönköping University, Jönköping, Sweden; Department of Health Sciences, Faculty of Medicine, Lund University, Lund, Sweden

## Abstract

Research examining Deaf older adults’ needs or experiences of old age care is scarce. This study aims to describe how Deaf older adults, supported by Swedish old age care, experience interactions and participation in everyday life and in social care situations. This study gives voice to Deaf older adults whose perspectives are rarely represented in research. To minimize misunderstandings, the qualitative individual interviews with Deaf older adults were conducted by Deaf signing research assistants. The results show that Deaf older adults face communication barriers due to a lack of shared language with their old age care staff, which may also pose health risks. Yet, despite the absence of sign language communication, participants described communication with those who help them as sufficient for basic needs, albeit limited. Furthermore, restricted community mobility contributed to sparse social contacts with Deaf peers, leading to social isolation and reduced social well-being.

## Deafness and sign language

There are about 70 million Deaf sign language users globally, and approximately 30,000 individuals use Swedish Sign Language (hereafter defined as sign language) (Region Stockholm, 2024). Deaf sign language users include people of all ages; however, more detailed data on specific age cohorts are lacking.

The words deaf and Deaf have two different meanings and are therefore spelled with a capital letter or a small letter. To be deaf (with a small letter d) is defined as having a hearing loss using spoken language. To be Deaf (with a capital letter D) refers not only to audiological status but to membership in a cultural and linguistic minority that uses sign language as its primary means of communication (Hiddinga & De Langen, 2019; Lesch et al., 2019). The general population, including old age care staff, often incorrectly understands Deafness solely as a hearing disability, overlooking its linguistic and cultural dimensions (Sadler et al., 2001). The largest group of sign language users is people born Deaf or with acquired Deafness early in life (Region Stockholm, 2024; Sadler et al., 2001). There are also hearing sign language users being part of Deaf culture; however, this group is not within the focus of the current study.

For Deaf individuals in Sweden, Swedish Sign Language is the native and most frequently used language (Region Stockholm, 2024). Swedish Sign Language is a distinct language with its own grammar, vocabulary, and syntax and is not equivalent to spoken Swedish (Region Stockholm, 2024; Sadler et al., 2001). Consequently, Deaf individuals experience communication barriers similar to those faced by other linguistic minority groups. Differences in grammatical structure increase the risk of misunderstandings when communicating in written Swedish (Sadler et al., 2001). Several studies have found that a large group of Deaf older individuals has limited reading skills (Region Stockholm, 2024; Sadler et al., 2001) and lower health literacy across written, spoken/lip-reading, and sign language forms modalities compared to the general population (McKee et al., 2015; Pollard & Barnett, 2009).

These challenges can probably partly be explained by the limitations in school education for this group when they were young. Their schooling often emphasized spoken language and lip-reading rather than sign language. As described in a Dutch discussion paper “the history of oralism … left many elderly deaf people embarrassed about their unruly writing” (Hiddinga & De Langen, 2019, p. 679). There is, of course, an individual variation, and it should be noted that many Deaf people are bilingual, using Swedish Sign Language as their first mother tongue and written Swedish as a second language.

As current life situations are influenced by past experiences, a life course perspective (Tornstam, 2018) supports the understanding of Deaf older adults’ current situation in the present study. This perspective emphasizes that, to understand an individual’s life today, it is necessary to consider their historical and biographical context, such as experiences of schooling and stigma. Life course perspectives have been widely applied in gerontological research in general and with a focus on communication (Davis et al., 2016). Deaf older adults share both collective and individual experiences from their life course that differ from those of their hearing peers. These experiences have formed them as individuals but also as part of a marginalized minority group. It is therefore important to include the perspective of Deaf older adults in research and to investigate how they experience everyday life aspects (Lesch et al., 2019).

## Deafness and aging

As part of natural aging, there are several changes in bodily functions, including hearing, vision, hand and finger dexterity, and cognition. These changes may impact communication and social and psychological well-being (Saadeh et al., 2020). These age-related changes also occur among Deaf older adults. Research has shown that ageing may affect the ability to communicate in sign language, for example, by making it more difficult to retrieve signs, reducing vocabulary, and impairing spatial abilities (Corina et al., 2020; Luna et al., 2021; Rantapää & Pekkala, 2016). Age-related visual impairments may further hinder communication by limiting the ability to perceive facial expressions, body language, and signed communication. Taken together, aging processes affect communication in multiple ways and place Deaf older adults at increased risk of exclusion and isolation. Consequently, Deaf older adults are at higher risk of loneliness, depression, mental distress, and reduced physical and psychological quality of life compared with their hearing peers. (Fellinger et al., 2005; Werngren-Elgström et al., 2003, 2006). For Deaf older adults receiving home care, additional communication barriers arise due to care staff’s limited knowledge of sign language and Deaf culture. These barriers contribute to lower levels of independence in communication-based activities and other activities of daily living (ADL) compared with hearing peers of the same age (Werngren-Elgström et al., 2005).

## Old age care support

Like the general population, many Deaf older adults receive support from old age care. In Sweden, such support should be person-centered (Andersson, 2013; Swedish Association of Local Authorities and Regions, 2024). Support may include ADL, such as hygiene and dressing, as well as Instrumental ADL (IADL), such as cleaning, grocery shopping, and accompanied walks. These interventions are integral to everyday life and are often intimate in nature, requiring sensitivity to individual preferences regarding, for example, food and clothing.

Person-centered care relies on interaction and communication. However, interactions between Deaf older adults and their formal carers are described as complex situations due to communication difficulties (Iezzoni et al., 2004; Lesch et al., 2019; Sheppard, 2014) due to the care staff lacking knowledge of sign language. These difficulties negatively affect communication, information exchange, trust, and the involvement of relatives, as experienced by both Deaf individuals and care staff (Iezzoni et al., 2004; Sheppard, 2014).

Previous research on home care for Deaf older adults with cognitive impairment, such as dementia, has demonstrated the importance of sign language for facilitating involvement in everyday decisions, promoting emotional expression, and creating a calming effect. Consequently, staff with sign language skills are of particular importance, yet remain scarce (Ferguson-Coleman et al., 2014; Rantapää & Pekkala, 2016; Rantapää et al., 2021, 2023). Research from the Netherlands has shown that residential care within a fully signing community can substantially reduce communication barriers (Hiddinga & De Langen, 2019). However, to the best of the authors’ knowledge, there is a lack of research considering Deaf older adults and their needs and/or experiences of old age care in ordinary homes or in residential care where nonsigning staff is the norm.

For all individuals in an aging population, contact with old age care increases due to age-related changes in the body and cognition. It is uncommon for care staff to know sign language, and many staff members also have a native language other than Swedish. In turn, this leads to misunderstandings between older adults and their staff but importantly also to social isolation, and restrictions in independence for Deaf older adults (Sadler et al., 2001).

## Aim

The aim of the current study is to describe how Deaf older adults in Sweden, that need old age care, experience (1) support from ordinary home care staff and (2) communication in everyday life situations in general, including communication with home care staff.

## Author positionality

Three of the authors, who are not researchers, are Deaf native sign language users (Å.G., L.J., and S.A.), and the other four authors (E.K., Y.M., S.K., and S.F.) are hearing researchers, nonfluent in sign language with basic knowledge about the Deaf community. The Deaf project coordinator of the mobile home care team (Å.G.) was the contact person of the two Deaf assistant nurses supporting the project with recruitment and project information. The two research assistants (L.J. and S.A.) conducted all interviews in sign language. The fact that they are part of Deaf culture and native sign language users gave them a unique possibility to understand the responses and ask relevant follow-up questions. All authors were involved in all parts of the study, from design and planning to reviewing the manuscript.

## Methods

### Design

To understand the experiences and needs of Deaf older adults, qualitative interviews were conducted. Ethical approval was obtained by the Swedish Ethical Review Authority (20200421, DNR: 2019-06372 and 20221017; DNR: 2022-05310-02). To bridge communication challenges related to implementation of informed consent and data collection (Paramasivam et al., 2021), we relied on Deaf and native sign language assistant nurses and research assistants to convey the content of the information letter to the participants.

### Participants

Six Deaf older adults (mean age 80 years) using Swedish Sign Language as their first language participated in the study and were recruited by convenience sampling (Golzar et al., 2022), supported by a nongovernmental organization for Deaf older adults. A brief overview of participant characteristics is presented in Table 1. All participants lived in the same county of Sweden and had support from either home care or residential care, by either the municipality in which they reside or a private care provider. Five participants were community-dwelling and had daily old age care, and one participant lived in a residential home for older adults. As the participants belonged to a minority group, no further demographic details are reported, as this could compromise confidentiality and risk identification.

**Table 1:** Participant information.

Participant ID	Gender	Age	Number of interviews
1	Female	73	4
2	Female	84	4
3	Male	88	4
4	Male	77	3
5	Female	77	1
6	Male	83	1

### Data collection

#### Interview guide

Prior to the data collection, a semi-structured interview guide was developed. The interview guide consisted of the topics: (I) support needed in everyday life activities and social life, (II) how the older adults communicated in everyday life situations and specifically with non-sign language users, (III) experiences of support from ordinary nonsigning care staff, (IV) experience of safety and security, and (V) suggestions for changes and improvements in the areas discussed. The interview guide was developed by all authors, including the experienced researchers and the Deaf authors. Through this process, an interview guide, relevant in sign language and in a Deaf culture context, was developed. This thorough work prior to the interviews increased language understandability and the relevance and trustworthiness of the study (Elo et al., 2014).

#### Interviews

All 17 interviews were conducted by native Deaf signing interviewers to avoid the need for additional interpreters. This approach was chosen as previous literature describes that Deaf older adults are often uncomfortable signing with hearing people or using their voice with unfamiliar individuals (Rantapää et al., 2023). Another factor for using Deaf signing interviewers was that their knowledge about Deaf culture would facilitate posing relevant follow-up questions.

A process consent approach was used. Before starting each interview, the research assistants (Deaf and using sign language) repeated the information about the study aim and the informed consent. The information was given in written format and sign language format. Written informed consent was given prior to the interviews and upon possibility for the participant to ask questions.

The interviews were conducted in Swedish Sign Language, in the participant’s home, by a Deaf research assistant, and the interviews took between 8 and 54 min (mean 22 min). The interviews were conducted across four different occasions (i.e., autumn 2022, spring 2023, autumn 2023, and spring 2024) for four of the participants. Two people participated in only one occasion each and then withdrew from the study due to health issues. Two Deaf research assistants (both care professionals) conducted the interviews—the first one conducted the first two series of interviews (i.e., autumn 2022 and spring 2023), while the second one conducted the last two series of interviews (i.e., autumn 2023 and spring 2024). All interviews were video-recorded, transcribed, and translated into written Swedish by the respective interviewer.

### Data analysis

#### Transcription

The video recordings were transcribed orthographically from sign language into written Swedish by the research assistants. During transcription, the research assistants reflected on the transcripts to confirm that they were adequately translated to support trustworthiness.

#### The steps of the content analysis

The transcribed interviews were analysed using content analysis according to the methodology described by Lindgren et al. (2020), as well as Graneheim et al. (2017). All analyses were conducted via the software NVivo version 14. Initially, meaning units relevant to the study aim were identified. The meaning units were then condensed and coded manifestly and closely to the text. Based on the codes, seven subcategories were interpreted. The subcategories were, in the next step, abstracted into three main categories (see Table 1). No joint overall theme was found or identified.

#### Analysis process, reflexivity, and credibility

The analysis process started with two authors (E.K. and S.F.) individually reading and analysing the data from the first round of interviews. After the first round of analysis, the initial results were then discussed by the two authors with the intention to try out the coding and our data matrix and ensure credibility and trustworthiness of analysis. E.K. has basic knowledge about Swedish sign language and Deaf culture and has a research background in audiology, gerontology, and disability research, and these valuable insights support the analysis and interpretation of findings. Similarly, S.F. has deep knowledge of gerontology. Next, the following three rounds of interviews were analysed sequentially. By analyzing one layer of interviews at a time, it became visible that the number of new codes decreased for each round of interviews, minimized the risk of category overlaps, and indicated sufficient category broadness and saturation; all parts strengthened the credibility and trustworthiness of analysis (Elo et al., 2014).

All categories were developed inductively from the full dataset, and all participants contributed to each category. When all interviews were coded, the authors discussed discrepancies identified in the analysis concerning both coding and interpretation. In a few cases, when transcripts were unclear or could be interpreted in several ways, the researchers consulted the Deaf research assistants for clarification. This was also a strategy to retain quality and trustworthiness and minimize bias in the analysis. The final results represented the full transcripts and strengthened the confirmability of the analysis. As a final step, the full research team reviewed and discussed the analysis and preliminary findings to ensure accurate reporting, interpretation, and overall trustworthiness.

The quotes displayed in the manuscript were translated from Swedish transcripts to English, mostly word by word, but when needed, they were adapted to make sense in the English language while remaining true to the original content.

## Results

The analysis resulted in three categories, together including seven subcategories, all displayed in Table 2.

**Table 2:** Overview of the categories and subcategories.

Categories	Subcategories
Independence and decision-making are restricted	Dependent but wishing to be independent. Being dependent in decision-making Security and trust vary by situation and person
At risk of social exclusion	Social engagement is important but challenging to uphold with limited community mobility. Lack of communication or communicating in other ways
Communication with home care or residential care staff	Communication difficulties with staff Continuity is important.

### Independence and decision-making are restricted

The first category was concentrated around a wish of being and staying independent and not receiving more care than needed. It also included a wish to be able to remain in their ordinary homes for as long as possible. Decision-making in everyday situations was found to be challenging as the lack of signing home care staff made it hard to communicate needs and wishes. This led to feelings of helplessness and insecurity.

#### Dependent but wishing to be independent

The participants expressed a wish to be independent. Nevertheless, their stories showed dependence on relatives like children or grandchildren. They all wished to stay in their ordinary homes for as long as possible and, as one of the female participants (P2 = participant 2) expressed, “I prefer to do things my way, I feel strong.” All the participants expressed a need to receive support concerning ADL, e.g., preparing meals or coffee, taking medications, taking care of hygiene, or changing clothes, as well as IADL including cleaning, laundry, and grocery shopping. They also wanted to receive support only with things they could not manage themselves, and one of the men (P3) concluded: “I don’t want to be considered spoiled, if I ask for too much help.”

#### Being dependent in decision-making

The participants’ overall experience was of not being involved in decision-making, both concerning major life decisions and everyday decisions in old age care situations. They lacked involvement in the decisions about what support to receive and how often. All participants felt that this had been decided by the staff and that they were rarely asked what kind of support they needed daily. This was true for the participants who received support in ordinary homes as well. The one participant in residential care experienced that it was possible to influence the support, although it was largely routinized. For participants with children, relatives often initiated and managed decisions on their behalf, for example, regarding housing, healthcare contacts, or personal finances. One woman (P1) exemplified: “My son looked for an apartment and found this one. He said it was nice, and I moved here as he told me to do so.” Most participants were worried about the economy, as they never had much money. And the same woman (P1) said: “I had to resign home care services for economic reasons. And I also want to manage myself. I can do laundries and everything myself.”

All participants were dependent on others to forward information, complaints, and questions to the staff, managers, and municipality representatives. Exemplified by one woman (P5): “No one has really told me how home care services work, or what I can ask for.” Written information from the municipality was difficult to understand, and as staff were unable to explain it in sign language, participants relied on relatives or other Deaf individuals for support. One man (P6) expressed: “I read and read but then I must wait for my son to come here. Then I show him the information or letter and ask him what it means. He reads the information and keeps in contact with the authorities.”

Decisions concerning participation in everyday activities such as food choice, preferred support from staff, or leisure activities were experienced as challenging. Participants preferred to get support from people they knew or could communicate with. Although they were able to make some decisions, limited access to information made decision-making more difficult. For minor, everyday decisions, relatives were less involved, and participants described that care staff did not consistently include them in these decisions. One of the women (P5) explained: “Berth [fictive name of a staff member] came in, but he doesn’t know any sign language. I just had to give up.”

#### Security and trust vary by situation and person

All participants reported feeling safe in their current homes. Safety alarms and doorbells with flashing lights contributed to the sense of safety. However, all participants felt even safer when staff members used sign language, even when using just a few signs.

One of the participants, while in need, did not trust the hearing staff at all and did not want to receive their support. One woman (P5) claimed she had asked her municipality for a Deaf home care staff: “They promised to look for a Deaf person, but I haven’t heard from them since.” Feelings of stigma were described when participants attempted to communicate using their voice or when sharing common spaces with hearing peers. One woman (P1) expressed how she did not mind using her voice when she met the same staff repeatedly: “They are used to my voice.” For others, the feelings of embarrassment of using their voice were so extensive that the participants refused to visit common spaces and preferred isolation, even though it led to loneliness. A woman (P2) who lived in a residential care facility for older adults explained: “I don’t often go to the [facility] restaurant, but when I do I write a note [telling what I want to eat] and leave it to them. I let them know that I want to bring the food up to my apartment, and then they give me a box… When my son visits, I eat in the restaurant.” However, in the company of a relative or another signing person, participants felt more confident engaging in shared spaces.

### At risk of social exclusion

The second main category of the results consisted of three subcategories and contained experiences concerning social engagement and its importance for well-being. Maintaining social engagement was challenging due to mobility limitations, which restricted opportunities to meet Deaf friends and family.

#### Social engagement is important but challenging to uphold with limited community mobility

Social engagement was of importance to all participants. Some were regularly visited by family and friends or engaged in regular video calls from relatives, while others were isolated, having very limited contact with others. Participants described social life as one of the aspects they missed most. All participants expressed sadness at losing their activities and contact with Deaf friends. Due to communication barriers and mobility difficulties, all but one participant needed support to be able to go out. Going out independently was described as essential for meeting Deaf peers. None of the participants were able to attend the Deaf club anymore due to transportation difficulties and physical inaccessibility of the premises. The deaf association’s rented premises have a chairlift, with an upper limit of transporting 100 kg, and thus, access was restricted for deaf people who weigh more than that. One woman (P1) concluded: “I wish the Deaf club could move down to the first floor, so I can get in with my wheeled walker.” More places to meet other Deaf older adults and a residential home for Deaf people were also wished for by participants.

They described a need for support to be able to go out not only to take a walk but also to have company when shopping or going to health care appointments. It was stated that the old age care services prioritized the ADL and IADL tasks and rarely had time to support social participation. One of the women (P5) illustrated a common wish among the participants: “I would like the staff to follow me to shops and restaurants, but that never happen. Only [mention a name of a friend] and I go to a café, but when she leaves me here, I’m lonely again.” These experiences led to not only loneliness but also feelings of missing out on taking part in social activities. However, the participants were thankful for the support they received and did not wish to seem too demanding on staff or relatives. Participant 5, when asked if she had communicated the wish illustrated above, said: “I have not contacted them. I don’t dare to.”

#### Lack of communication or communicating in other ways

Participants mentioned using technology for making video calls with friends and family to compensate for limited social interactions. While that brought social connections, one of the women (P2) exemplified: “I feel lonely nevertheless, I wonder what to do. I call my friend using video and she tells me what she has done and that she had visits from her son. Good for her, but my own son doesn’t visit. I think a lot about that. I don’t understand [sighing].”

The participants tried to find things to keep them busy at home to compensate for loneliness, and most of the participants watched a lot of television. One participant slept a lot as a way of trying not to think about loneliness. All participants reported having no one with whom they could communicate in sign language on a daily basis. A few participants felt confident using their voice once they became familiar with others. One man (P6) living in a residential housing said: “Many men live here. I can talk to them. Thanks to that I learnt to speak when I was a child.”

### Communication with home care or residential care staff

The third category focused on communication with care staff. As participants spent most of their time at home and required daily support, care staff were often the only people they encountered. However, the lack of sign language knowledge among staff limited communication. To compensate for these limitations and obstacles, other means of communication were accommodated and, even if it was experienced as better than nothing by the participants, there were still limitations. Continuity concerning staff was highlighted as an important factor as it was experienced as easier to communicate with well-known staff members.

#### Communication difficulties with staff

All participants talked about communication with the ordinary home care and residential care staff. They all expressed barriers and that most communication was spoken/lip-read or written on a piece of paper, both accommodations made by the Deaf as the staff had no knowledge of sign language. The communication between the participant and the staff was therefore limited. There was no room for small talk or to ask questions. One man (P4) suggested: “They [the staff] could sign up for a sign language course,” and one woman (P5) described how her age-declining vision brought complications: “In the beginning I thought it worked well…but over time I discovered difficulties in reading lips.”

Participants reported better communication with some staff members than others and preferred assistance from those they could understand more easily. Staff members who knew even a few signs were favoured. When spoken communication/lip-reading did not work, short notes were written on paper, again an accommodation made by the Deaf older adults: When the staff come here and I don’t understand them, I ask them to write with paper and pen.” (P2).

In some cases, communication was described as completely ineffective, leading to frustration and helplessness. One man (P3) stated: “Well, he [a staff member] speaks rather unclearly, so it is hard to lip-read. Then I feel helpless. The other person, the woman, talks clearly and then I understand. Even though there are staff members not using sign language, the [basic] communication works.” The participants also experienced that staff members not having Swedish as a first language, which was common, were extra hard to understand. One man (P3) said: “I have to endure the situation.” Similar acceptance of communication as sufficient was confirmed by the other participants, describing communication as sufficient to get simple tasks done, while misunderstandings occurred on a regular basis, concerning, for example, grocery shopping and medications.

#### Continuity is important

Continuity of care staff was something all participants preferred. Familiarity with staff made communication easier and reduced embarrassment associated with using one’s voice. For the participant living in residential care, weekend staff (often also temporary) were particularly difficult to communicate with, resulting in frustration and reduced willingness to accept support during weekends. Negative emotions were also expressed when well-known staff or staff with some sign language left their position, something that several participants had experienced repeatedly. One man (P3) who had moved from a short-term residential care facility to a permanent facility described: “It took me nearly 6–7 months before I felt safe here.”

## Discussion

This study demonstrates that Deaf older people experience interactions and participation in everyday life situations, including social care, in diverse ways. While some participants reported relatively rich social interaction, others experienced profound loneliness. However, they all shared experiences in relation to interaction with hearing staff, communication with other Deaf people, and limitations in self-determination.

Participants appreciated the possibility to get support from old age care staff, as they needed assistance with ADL and IADL. However, they consistently described communication errors as frequent and as substantially limiting their ability to express needs, preferences, and concerns. Communication difficulties also led to reduced trust in care staff and indicated that care was not consistently aligned with participants’ needs or preferences. Despite these shortcomings, the participants expressed gratitude for receiving support and did not wish to appear demanding or to request additional support. They described communication as sufficient to get certain basic tasks done yet insufficient for meaningful interaction or shared decision-making. However, it is problematic that the Deaf older adults are not supported in line with the principles of person-centered care (McCormack et al., 2021). Within Swedish health care, an ethical and person-centered approach should be applied (Andersson, 2013) and one important part of this approach is the right to self-determination, defined as everyone’s right to make informed decisions. Self-determination requires access to information and the ability to make informed decisions, both of which depend on effective communication (The Swedish National Council on Medical Ethics, 2024). Previous research suggests that nurse assistants in old age grapple to prioritize older peoples’ exercise of autonomy (Kjellström & Sjölander, 2014). The present findings illustrate that Deaf older adults are often not afforded this right, particularly due to communication barriers.

Moreover, it remains an open question whether the service that they receive fully meets the directives of equality and active social participation expressed in the current Swedish social service act (2001). This is particularly relevant in light of the revised Social Services Act introduced in 2025, which aims to strengthen prevention, equity, and accessibility in social care. The experiences described by participants suggest that current services fall short of these ambitions.

From an outsider perspective, it appears that participants accepted support in intimate and personal matters despite insufficient communication. Not all other older people would accept that, and likely protest or at least comment on said flaws or issues when given the possibility to give feedback. It is possible that the participants are content with not being in contact with hearing peers and thus lacking awareness of the differences in services received. However, it is perhaps more likely that their viewpoints originate from experiences throughout their lives. In line with a life course perspective (Tornstam, 2018), the collective and individual history of the Deaf culture impacts Deaf older adults’ current experiences of everyday life and communication. For example, Deaf older adults have often been required to adapt to hearing norms rather than having their own linguistic and cultural needs accommodated (Eriksson, 1993). Consequently, the participants in our study were not used to having a choice and accepted their situation and did not demand sign language communication. This is also reflected in the result where the participants did not want to trouble anyone, not asking for extra support or demanding proper communication. Such acceptance is common also among older adults in general (Teo et al., 2022) in Sweden, as well as internationally (Baeriswyl & Oris, 2023; Nakamura et al., 2022).

Moreover, the participants experienced it harder to communicate in later life due to decreased vision. That age-related changes may impact the ability to use sign language is displayed in previous research (Corina et al., 2020; Luna et al., 2021; Rantapää & Pekkala, 2016). However, the participants found it more difficult to lip-read than to use sign language. Consequently, this very common way of communicating between the Deaf and hearing becomes less feasible in later life. Given that many Deaf older adults also have limited proficiency in written Swedish (Region Stockholm, 2024), reliance on written notes as the primary mode of communication is inadequate and increases the risk of misunderstandings. The frequent misunderstandings described by participants indicate that current communication practices are neither appropriate nor sustainable.

Our findings also display Deaf older adults’ dependence on hearing relatives to be able to manage communication surrounding personal finances, contact with authorities, etc., also noted by Eriksson (1993). The concept of audism, first described in 1975 (Humphries, 1975), shed light on this and similar issues (Eckert & Rowley, 2013). Historically, Deaf children were often sent to boarding schools and were often forced to adapt to spoken language environments, sometimes resulting in long-lasting feelings of stigma and embarrassment (Pärsson, 1997). In the current study, most participants felt embarrassed to talk/use their voice around hearing people. This is an example of the stigma also placing hearing and spoken language as the societal norm (Da Silva et al., 2023). As most people use spoken language and the societal structures and services are built on spoken language, Deaf older persons’ contact with service providers, authorities, etc., becomes restricted. On the same note, some participants did not feel confident enough to eat in the shared dining area for older people. This audism stigma is hidden but has a major impact on social life and well-being for Deaf older people. Research such as the present study is therefore essential to challenge hearing-centred norms and to promote more inclusive care practices.

Moreover, the Deaf older adults’ personal decisions were often made by relatives or the staff. With services based on sign language, these limitations would not appear. According to the Swedish National Board of Health and Welfare (2015), older adults in Sweden have the right to access care partly or fully in their own language and culture. The present findings suggest that this right is not adequately realized for Deaf older adults. Structural challenges, including staff shortages and high turnover in old age care, have been identified as contributing factors (Swedish Agency for Health and Care Services Analysis, 2024). This conflicts with Agenda 2030 and the goals of *Reduced Inequality* and *Good Health* (United Nations, 2015).

Communication with other or among Deaf people and participation in Deaf clubs were considered essential among all participants, in line with previous literature (Eriksson, 1993; Pärsson, 1997). Audism and hearing as the norm in society explain this consideration (Eckert & Rowley, 2013). For the mobility-impaired participants of the current study, it was not possible to access the Deaf clubs anymore and, as many of their peers have passed away, their social network consequently has decreased. This is true for older adults in general (Fristedt, 2012); however, when applying a life course perspective, it is found that Deaf older adults, because of a history of exclusion, stigma, and dependence, are at greater risk of total lack of communication and total isolation than their hearing peers. The study results confirm this as most participants missed communication with Deaf peers, and the communication with home care/residential care staff was almost nonexisting. Similar situations can be seen for immigrants with other native languages. However, Deaf individuals using Swedish Sign Language have clearly limited possibilities to reach arenas (Fristedt, 2012) where their native language is used, with likely implications for their social participation.

### Future recommendations

The results of the study indicate that signing home care and residential care staff is of importance for Deaf older adults. Currently, only a limited number of residential care facilities for Deaf people exist in Sweden, and due to geographical distance, moving into such facilities often requires relocation to another part of the country. This presents a significant barrier, particularly for individuals who wish to remain close to family and established social networks. In the Netherlands, Deaf Culture and sign language-based living environment with different levels of care, and Deaf older adults with varying needs, has been a success (Hiddinga & De Langen, 2019). Although no comparable model currently exists in Sweden, several participants expressed interest in such arrangements. While relocation may not be desirable or feasible for all, this model could be explored as an option for those willing to move. As an alternative, a Mobile home care team consisting of Deaf signing nurses has been implemented and tested in a municipality of Sweden (Karlsson et al., 2025). The Deaf individuals trying this model were content, but the long-term financing remains uncertain, and national guidelines would be needed because municipal resources differ. The present study also highlights the need for broader efforts to increase sign language competence among care staff. National initiatives aimed at recruitment, education, and training are necessary to improve communication accessibility in old age care. Recruiting Deaf assistant nurses may represent a viable strategy, particularly if outreach efforts target young Deaf individuals early and encourage entry into care professions. Such initiatives could contribute to more sustainable, inclusive care models that better meet the needs of Deaf older adults.

### Strength and limitations

The study is one of the few investigating how Deaf older adults experience interactions in everyday life situations and in social care situations. As such, direct comparison with previous studies is restricted.

Twelve Deaf older adults within the chosen geographical context were invited to be part of the study, and six of them agreed to take part in the study. The small sample size (*n* = 6) can probably be explained not only by the resources of energy needed to participate in a study but also by Deaf people historically being excluded from studies and having less trust in unknown nonsigning people (Lesch et al., 2019; Rantapää et al., 2023; Werngren-Elgström et al., 2005). In qualitative research, the sample size is often smaller as the focus is to collect rich and in-depth data and not statistical power (Patton, 2015). To compensate for the low sample size and enhance trustworthiness, the interviews were conducted on several occasions. Over the series of interviews, trust was built, which contributed to more detailed and reflective accounts. In the end, almost no new information was added in the final round of interviews, which may be an indicator of a sufficient amount of data. However, further studies are necessary, including a larger sample size.

## Conclusion

The aim of the study was to describe how Deaf older adults experience support from home care staff and residential care staff. The experiences of Deaf older adults varied, but all experienced communication barriers due to the absence of sign language–competent staff. Deaf older adults and the old age care staff lack a shared language and face communication difficulties on a daily basis, clearly restricting deaf older adults’ possibilities to express their needs and preferences. These barriers increase the risk of misunderstandings, dependence on others, and social isolation. The old age care staff need more concrete guidance on how to meet the needs of Deaf older adults in daily care. To meet the requirements stated in the new Swedish social service act (2025), guidelines addressing communication accessibility and linguistic inclusion are required. Moreover, interventions including home care or residential care staff with sign language competence would likely benefit Deaf older adults in several ways. By improving communication, supporting decision-making, and enabling participation, such interventions have the potential to promote independence, quality of life, and well-being.

## Data Availability

Deidentified data are available upon request, in line with ethical approval.

## Clinical trial

Clinical trial number: not applicable.
